# Antibacterial Efficacy of Benefect™ Botanical Disinfectant in Comparison with Sodium Hypochlorite and Chlorohexidine Against Multiple Endodontic Pathogens: An Ex Vivo Study

**DOI:** 10.3390/dj13020087

**Published:** 2025-02-18

**Authors:** Sarmed Toma, Joseph Ferracciolo, Mazin Askar, Eric Krukonis, Susan Paurazas

**Affiliations:** 1 Graduate Endodontics, University of Detroit Mercy School of Dentistry, Detroit, MI 48208, USA; tomasa@udmercy.edu (S.T.); askarma@udmercy.edu (M.A.); 2 Division of Integrated Biomedical Sciences, University of Detroit Mercy School of Dentistry, Detroit, MI 48208, USA; ferracjo@udmercy.edu

**Keywords:** essential oil, irrigation, disinfection, endodontic pathogens, biofilm, *E. faecalis*

## Abstract

**Background/Objectives**: Natural antibacterial agents, such as essential oils, can potentially be used for endodontic disinfection with less toxicity than other available irrigants such as sodium hypochlorite (NaOCl) and chlorhexidine (CHX). Benefect^TM^ is a formulation of essential oils with broad antibacterial spectrum efficacy. This study aims to compare the antibacterial efficacy of Benefect^TM^ to 6% NaOCl and 2% CHX irrigant solutions against multiple endodontic pathogens. **Methods**: The study utilized 100 extracted human single-canal permanent teeth. Samples were decoronated, instrumented, and autoclaved. The teeth were infected with *Streptococcus mutans*, *Enterococcus faecalis*, *Actinomyces naeslundii*, or *Porphyromonas gingivalis* for 6–24 h. The teeth were divided into four groups according to the irrigant solution used. Contact with each irrigant was maintained for 12 min. The antibacterial efficacy of each treatment was calculated relative to viable bacteria recovered after saline treatment. Statistical analysis was performed using Student’s *t*-test. **Results**: All *S. mutans* samples treated with NaOCl, CHX, and Benefect^TM^ showed a complete absence of bacterial colonies when compared to saline (>99.9% killing). The *E. faecalis*, *A. naeslundii*, and *P. gingivalis* samples treated with all tested irrigants showed at least 99% antibacterial killing activity. There was no statistical difference in killing between these three antimicrobial treatments. **Conclusions**: Benefect^TM^ botanical disinfectant has comparable antibacterial efficacy to NaOCl and CHX against *S. mutans*, *E. faecalis*, *A. naeslundii*, and *P. gingivalis*.

## 1. Introduction

Microorganisms are the major determinant in endodontic infection [1]. Endodontic infections occur when the root canal system is in contact with the oral environment and concurrently when there is a decrease in the host’s local immune response [2]. The microorganisms are enclosed within the intra-radicular area following a carious lesion or a traumatic injury to the coronal tooth structure. However, if not treated, the pathogens and their by-products emerge from the apical foramen to the periradicular tissues [3]. Endodontic infections can be subdivided into three categories: primary, secondary, and persistent depending on the time when microorganisms enter the pulpal space [4]. Primary endodontic infections are caused by microorganisms involved in initial pulp invasion and subsequent colonization of necrotic tissues. Secondary endodontic infections are caused by microorganisms introduced into the root canal secondarily to clinical intervention. This can occur iatrogenically during operative procedures or later by coronal microleakage. Persistent endodontic infections are caused by microorganisms that are part of either a primary or secondary infection that resisted chemo-mechanical debridement procedures and survived within the nutrient-deficient environment of treated root canals. Because persistent and secondary infections remain clinically challenging to distinguish, they tend to be regrouped under the same pathological entity [5]. Primary infections appear to be dominated by 40–50 Gram-negative strictly anaerobic species of the genera *Fusobacterium*, *Prevotella*, *Porphyromonas*, *Tannerella*, and *Treponema* [5]. On the other hand, persistent/secondary infections seemed to harbor less diverse microbial communities composed of 10–20 taxa, mostly Gram-positive facultative anaerobes including species of *Streptococcus*, *Lactobacillus*, *Actinomyces*, and *Enterococcus* or the oral yeast *Candida albicans* [3,6,7]. One of the main objectives of endodontic treatment is the removal of inflamed pulp tissue and associated microorganisms. Since mechanical preparation procedures have been deemed insufficient, a disinfecting root canal irrigation solution is essential in order to achieve effective bacterial removal from the root canal system [8]. Ideal irrigation solution should have a broad antimicrobial spectrum, be efficient against obligately anaerobic and facultatively anaerobic microorganisms/biofilms, inactivate endotoxins, dissolve pulp tissue, avoid smear layer formation during instrumentation, and should not irritate the periodontal tissues [9]. Sodium hypochlorite (NaOCl) is the most commonly used irrigant in endodontics due to its antimicrobial effect and tissue-dissolving properties [9,10]. Previous studies confirmed the use of NaOCl against *E. faecalis* as a very potent root canal irrigant; however, its bactericidal efficacy is concentration-dependent, with higher concentrations resulting in better outcomes [11]. On the other hand, high concentrations of sodium hypochlorite, such as 5% or 9%, may result in the breakdown of the organic dentin matrix. Such concentrations may be caustic to periapical tissues, particularly if extruded out of the apical foramen [12]. Chlorhexidine (CHX) has been advocated as an irrigation solution due to its wide range of antimicrobial activity and its substantivity [13]. Some shortcomings of CHX include its inability to dissolve organic matter and its potentially toxic impact on periapical tissues [14,15]. Furthermore, it has been reported that CHX is not capable of penetrating deep layers of thick biofilms, thus having a higher bactericidal effect on early biofilms rather than mature biofilms [16]. As a result of limitations with current disinfecting irrigating solutions, there is increasing interest in the use of natural compounds in endodontic disinfection. [17]. Herbal compounds used in medicine have anti-oxidant, anti-microbial and anti- inflammatory properties, desirable properties for endodontic application. [18]. In this study, we assess Benefect™, a new formula of essential oils made from plant extracts being used as surface disinfectant, that may provide a more natural alternative that is antibacterial. Benefect™ has broad spectrum efficacy: bactericidal, virucidal, fungicidal, and tuberculocidal with a mechanism of action of disruption of cell membranes. Benefect™’s ingredients include thyme oil (0.23%), lemongrass oil (0.1–1.0%), biosurfactants, water, water ionizer. Benefect™ is classified under the EPA’s lowest toxicity rating allowed by law for all routes of exposure which include inhalation, ingestion, skin irritation, skin sensitivity, and eye irritation [19]. The aim of the study is to compare the antibacterial efficacy of Benefect™ disinfectant with 6% NaOCl and 2% CHX irrigant solutions against multiple endodontic pathogens: *S. mutans*, *E. faecalis*, *A. naeslundii* and *P. gingivalis.*

## 2. Materials and Methods

### 2.1. Specimen Selection and Preparation

This study was granted an exemption by the University of Detroit Mercy Institutional Review Board (#23-24-17, approval date: 15 August 2023) in accordance with Department of Health and Human Services (DHHS) Regulations for Protection of Human Subjects. One hundred intact, unrestored, non-carious, mature human single-rooted, freshly extracted teeth from adults for orthodontic or periodontal reasons were selected. Exclusion criteria included teeth with caries, fractures, resorption, or obliterated canal space. Teeth were cleaned and stored in 3% NaOCl at room temperature. Each tooth was examined radiographically in two planes to ensure the presence of a single canal. Teeth were decoronated at cemento-enamel junction (CEJ) using #557 surgical bur (Komet USA, Fort Mill, SC, USA) with a high-speed handpiece. Apical foramen enlargement of all samples was carried out up to International Organization for Standardization (ISO) size #20 using hand K files ISO size #8, #10, #15, and #20 (MANI, Takenzawa, Japan). During the process of instrumentation, all canals were irrigated with 5 mL of saline using a syringe with a 30-G side-vented needle (ProRinse; Dentsply-Maillefer, Ballaigues, Switzerland). The apex of each tooth was sealed with composite to prevent the leakage of irrigants and microorganisms. The external part of each tooth’s root was sealed using nail polish. After preparation, teeth were stored in a sterile saline solution.

### 2.2. Sterilization and Asepsis Control

Each tooth was autoclaved for 15 min at 121 °C and stored in sterile 1× phosphate-buffered saline (PBS) (Corning 21-040-CV, Manassas, VA, USA) for at least 48 h.

### 2.3. Root Canal Inoculation

The bacterial strains used in this study were *E. faecalis* (ATCC29212, ATCC, Manasses, VA, USA), *A. naeslundii* (ATCC12104, ATCC, Manasses, VA, USA), *S. mutans* (ATCC 25175, ATCC, Manasses, VA, USA), *P. gingivalis* (ATCC33277, ATCC, Manasses, VA, USA). These represent Gram-positive and Gram-negative species commonly recovered from infected root canals. Overnight cultures of *S. mutans*, *E. faecalis*, and *A. naeslundii* were grown in BHI at 37 °C (5% CO_2_ for *S. mutans* and *A. naeslundii*). *P. gingivalis* was grown overnight anaerobically in brucella broth with 0.5 µg/mL vitamin K and 5 µg/mL hemin. Overnight cultures of each species were pelleted for 10 min at 4500 rpm, washed once with 10 mL PBS, and re-pelleted for 10 min at 4500 rpm. Bacteria were then resuspended to a final concentration of OD_600_ = 1.0 in artificial saliva [20] with 0.5% sucrose (except *P. gingivalis*, which was resuspended in brucella broth with 0.5 µg/mL vitamin K and 5 µg/mL hemin). All teeth were inoculated with ~1,000,000 colony-forming units (CFU)/canal (10 ml of OD_600_ = 1.0 culture). Inoculated teeth were incubated overnight at 37 °C in 5% CO_2_ (*S. mutans* and *A. naeslundii*), 37 °C without CO_2_ (*E. faecalis*) or 37 °C anaerobically for 4–6 h (*P. gingivalis*) to allow the bacteria to adhere and penetrate the dentin.

### 2.4. Processing of Specimen Samples

**Figure 1:**
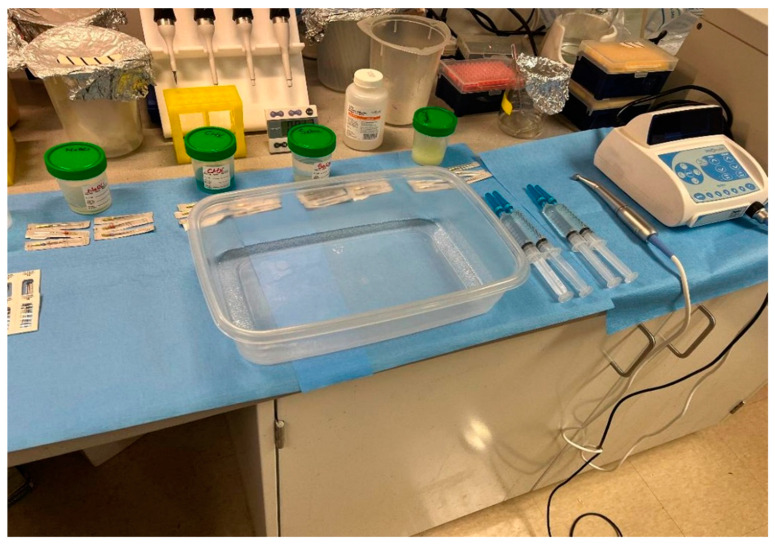
Experimental setup for tooth colonization, instrumentation, and irrigation. Included are an endodontic motor (ProMark, Dentsply Tulsa Dental, Johnson City, TN, USA), Protaper Ultimate F1, F2, and F3 rotary files (Dentsply Sirona USA, Charlotte, NC, USA), irrigant solutions NaOCl, CHX, Saline, and Benefect^TM^ placed in sterile cups; and a disinfected container where the instrumentation and irrigation of inoculated teeth took place.

All teeth were instrumented using Protaper Ultimate F1, F2, and F3 files (Dentsply Sirona USA, Charlotte, NC, USA). Between instrumentations, the canals were irrigated with 9 mL (3 mL/4 min) of the corresponding Benefect^TM^ (Sensible Life Products, ON, Canada), NaOCl (Pure Bright, ON, Canada), CHX (Vista Apex, Racine, WI, USA), or saline solution (Stericare Solutions, Haltom City, TX, USA) for 12 min, to ensure efficient killing by Benefect^TM^ [17]. At the end of preparation, each canal was supplementarily irrigated with 10 mL of sterile saline for 2 min to remove the remaining Benefect^TM^, NaOCl, and CHX (Figure 1).

### 2.5. Microbial Sampling and Bacterial Counting

Sterile paper points were used for each canal for post-instrumentation sampling. Paper points were maintained in the canal for 1 min and sterilely transferred to 0.5 mL of sterile PBS and vortexed for 10 s to release bacteria from the paper point. A total of 50 µL of a series of 10-fold dilutions was plated on Brain Heart Infusion agar (BHI) or Brucella Broth agar (BD Laboratories, Sparks, MD, USA #BD211086) supplemented with 5% sheep blood (Hemostat Laboratories, Dixon, CA, USA) 0.5 µg/mL vitamin K (Sigma-Aldrich, St. Louis, MO, USA #V3501), and 5 µg/mL hemin (Acros Organics/Fisher Scientific, Waltham, MA, USA #345960250) (BBA) plates for 2–7 days and colonies were counted. The efficacy of each chemical treatment was calculated relative to the number of colonies recovered after treatment with saline solution alone (negative control treatment). To demonstrate the ability of bacteria to colonize canals, some teeth were sampled without any chemo-mechanical treatment.

### 2.6. Liquid Killing Assay

Two days prior to the experiment, *P. gingivalis* cultures were prepared in 10 mL of brucella broth (BD Laboratories, Sparks, MD, USA #BD211088) supplemented with 0.5 µg/mL vitamin K and 5 µg/mL hemin (also known as BB+ media). Cultures were made anaerobically in pre-equilibrated 15 mL polystyrene test tubes (Falcon, Manassas, VA, USA #352025). One day prior to the experiment, cultures were diluted 1:10 into 10 mL of fresh pre-equilibrated BB+ media. On the day of experiment, OD_600_ measurements were taken from the subcultures and used to resuspend the *P. gingivalis* cultures to OD_600_ = 1.0 in sterile PBS to a final volume of 5 mL. A total of 450 µL of each treatment (0.9% Saline, 6% NaOCl, 2% CHX, Benefect^TM^) was transferred to sterile 1.5 mL microcentrifuge tubes (USA Scientific, Enfield, CT, USA). A total of 50 µL of the OD_600_ = 1.0 bacterial stock was added to the treatment tube and incubated at room temperature for 12 min. The tubes were then centrifuged at 7000 rpm for 5 min. A total of 450 µL of the supernatant was subsequently removed from the tubes and the remaining bacterial pellet was resuspended in 450 µL sterile PBS. The tubes were serially diluted (50 µL of bacteria into 450 µL of PBS) and 50 µL of each dilution was plated onto BBA. Plates were incubated in anaerobic gas packs (BD Laboratories, Sparks, MD, USA #BD260683) for nine days until complete growth appeared.

## 3. Results

**Figure 2:**
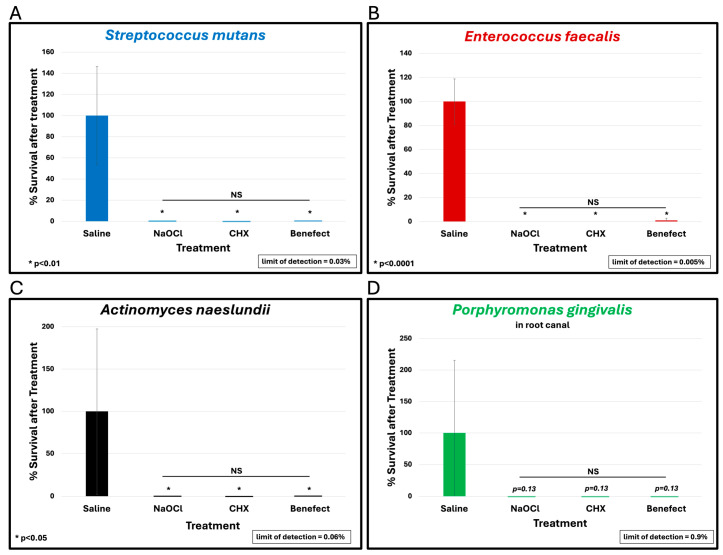
Benefect^TM^ is as effective as NaOCl and CHX in disinfecting root canals. Benefect^TM^ showed a complete absence of bacterial colonies when compared to saline when used against *S. mutans* (>99.9% killing) with no difference among all treatment groups (**A**). NS = no statistical difference between treatments. Similarly, Benefect^TM^ resulted in 99% killing of *E. faecalis* (**B**) or *A. naeslundii* (**C**) with no difference between all treatment groups. While *P. gingivalis* treated with Benefect^TM^ resulted in 99% killing (**D**), this did not reach statistical significance compared to saline (*p* = 0.13).

**Figure 3:**
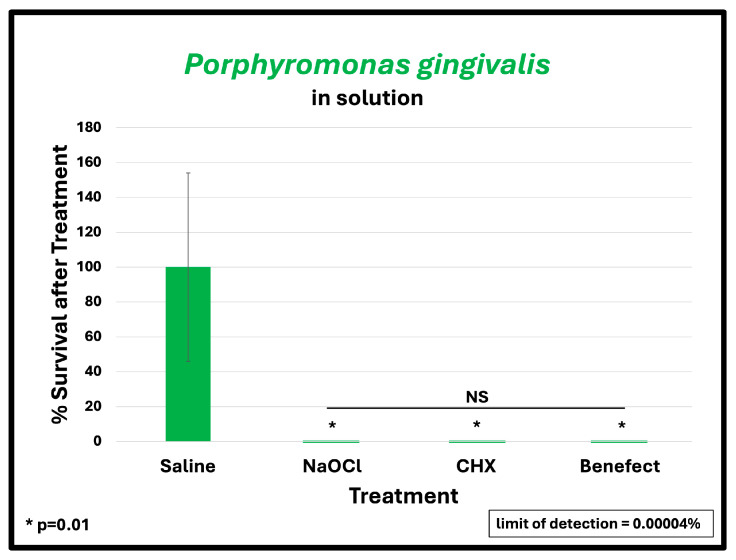
Benefect^TM^ is as effective as NaOCl and CHX in eliminating *P. gingivalis* in liquid-killing assay. Benefect^TM^ and other treatments all resulted in >99.9999% killing efficacy with no difference among all treatment groups.

To assess the antimicrobial effect of Benefect^TM^ compared to NaOCl and CHX, teeth were infected with different endodontic pathogens. Following colonization, each potential antimicrobial irrigant was assessed for root disinfection relative to saline (negative control). The level of bacteria in the teeth irrigated with saline was set to 100% and the relative levels of each bacterial pathogen after treatment with Benefect^TM^, NaOCl and CHX were determined. *S. mutans* samples treated with Benefect^TM^ showed a complete absence of bacterial colonies when compared to saline (>99.9% killing), similar to NaOCl, and CHX (Figure 2A). Treatment of both *E. faecalis* and *A. naeslundii* with Benefect^TM^ resulted in 99% killing, again with no statistical difference compared to NaOCl and CHX (Figure 2B,C). While treatment of *P. gingivalis* with Benefect^TM^, NaOCl, and CHX resulted in 99% killing, this result was not statistically different than saline, due to the fact that several teeth were poorly colonized by *P. gingivalis*, prior to chemical treatment (Figure 2D). Thus, to assess whether *P. gingivalis* is sensitive to the Benefect^TM^ treatment, we performed a liquid-killing assay, in the absence of teeth. Treatments of *P. gingivalis* with Benefect^TM^, NaOCl, and CHX all resulted in >99.9999% killing in a liquid-killing assay (Figure 3). Experiments were performed 2–3 times in duplicate (n = 4–6). Differences were assessed by Student’s *t*-test.

## 4. Discussion

This is the first study to assess the antibacterial efficacy of Benefect^TM^ as a potential endodontic irrigant solution, and the first study to assess the formulation of combined essential oils of thyme, and lemongrass. We found Benefect^TM^ is as effective as NaOCl and CHX in disinfecting the root canal space. The individual essential oils contained in Benefect^TM^ have shown antibacterial activity against endodontic pathogens. In an in vitro study comparing the antimicrobial efficacy of two types of endodontic sealers (zinc oxide with eugenol and zinc oxide with thyme oil [ZO + Th]) against *Staphylococcus aureus*, *Escherichia coli*, *Enterococcus faecalis*, and *Pseudomonas aeruginosa*, ZO + Th oil paste showed a higher antimicrobial effect against root canal pathogens [21]. An in vivo study revealed that thyme irrigant solution exhibits a significant reduction in *E. faecalis* [22]. In an in vitro study that evaluated the antibacterial efficacy of different concentrations of oregano oil, the results demonstrated that 1% Oregano Extract Solution (OES) and 5.25% NaOCl have the same antimicrobial activity against *E. faecalis* and 2% or 5% OES had more effective antibacterial action than 5.25% NaOCl [23]. Another in vitro study found that 0.2% oregano essential oil showed better antibacterial activity against *E. faecalis* when compared to 3% NaOCl and 2% CHX [24]. Lemongrass oil shows antimicrobial activity against *E. faecalis* and *Candida albicans* [25]. Lemongrass extract (*Cyombopogon citratus*) was proven to be more effective than sodium hypochlorite 2.5% in inhibiting the growth of *E. faecalis* bacteria [26]. In an in vitro study, the antibacterial efficacy of thyme, oregano, and lemongrass oils was evaluated separately in *E. faecalis* infected teeth with and without instrumentation. In the first processing (instrumentation with irrigation), thyme completely inhibited *E. faecalis* growth. In the second processing (without instrumentation, only irrigation), the results showed a complete clearance of *E. faecalis* by oregano, as well as by 5.25% NaOCl. Thyme was also highly effective, with only a few colonies observed on the culture medium after irrigation. Lemongrass also decreased the bacterial load by more than 75% [27]. Our study shows that the combination of the essential oils thyme and lemongrass in addition to instrumentation provides an antibacterial efficacy comparable to NaOCl and CHX [28]. These findings are in agreement with Nagy-Bota et al., 2021 [27], although in our study, the antibacterial efficacy of the essential oils was assessed as a combined formulation. In addition to *E. faecalis*, we assessed the killing of three additional endodontic pathogens. The findings of this study support Pedrinha et al., 2025 [29], who found that natural disinfecting agents propolis and copaiba oral resin acted similarly to conventional agents against a dual-species biofilm of *E. faecalis* and *S. mutans* as well as *S. oralis* and *A. naeslundii.* Future directions will focus on assessing the antibacterial efficacy of multiple organisms grown in biofilms associated with secondary/persistent endodontic infection, and the toxicity of Benefect^TM^ on periapical tissues. The limitations of this study include the use of single-rooted teeth. Complex canal anatomy in multirooted teeth with the presence of isthmus, fins and accessory canals may affect the antimicrobial efficiency of mechanical instrumentation and chemical disinfection. Another limitation is the use of paper point sampling where the accuracy of sampling and the absorbance rate may vary despite the controlled experimental conditions; also, contamination is possible during handling. We note that contamination was not an issue in our study. This study used monospecies biofilms with endodontic pathogens. While endodontic infections are polymicrobial, establishing balanced in vitro polymicrobial biofilms is challenging and often unsuccessful. Despite these challenges, building polymicrobial biofilms for treatment with Benefect^TM^ may be attempted in future experiments.

## 5. Conclusions

Benefect^TM^ is as effective as NaOCl and CHX in disinfecting the root canal space. Benefect^TM^ has the potential to be a natural alternative to conventional irrigation solutions.

## Data Availability

The data presented in this study are available upon request from the corresponding author due to privacy.
